# Implementation and first experiences with a multimodal mentorship curriculum for medicine-paediatrics residents

**DOI:** 10.1080/07853890.2022.2070661

**Published:** 2022-05-11

**Authors:** Lao-Tzu Allan-Blitz, Yannis Valtis, Michael Sundberg, Niraj Sharma, Elizabeth Petersen, C. Nicholas Cuneo

**Affiliations:** a Department of Medicine, Brigham and Women’s Hospital, Boston, MA, USA; b Department of Pediatrics, Boston Children’s Hospital, Boston, MA, USA; c Harvard Medical School, Boston, MA, USA; d Departments of Medicine and Pediatrics, University of Minnesota Medical School, Minneapolis, MN, USA; e Departments of Pediatrics and Medicine, Johns Hopkins University School of Medicine, Baltimore, MD, USA

**Keywords:** Mentorship curriculum, peer groups, medical residency, junior physicians, medicine-paediatrics residents, peer mentorship

## Abstract

**Introduction:**

Mentorship increases trainee productivity, promotes career satisfaction and reduces burnout. Beginning in 2016, our Medicine-Paediatrics residency program developed and implemented a longitudinal mentorship curriculum among trainees. We report initial experiences with that program and discuss potential future directions.

**Curriculum structure and method of implementation:**

We implemented and adapted a peer mentorship model and expanded it to include guest lectures and workshops centred around 13 core topics. Our expanded model included five longitudinal components: (1) peer mentorship; (2) virtual check-ins with residency leadership; (3) focussed didactics and workshops; (4) small-group dinners highlighting different career paths; and (5) dedicated faculty who pair residents with mentors based on common interests. We compared annual survey results on resident satisfaction with program mentorship, using chi-square and fisher’s exact tests to assess statistically significant differences pre- (2012–2016) and post-intervention (2016–2020).

**Results:**

We analysed 112 responses with annual response rate varying between 41.2% and 100%. Overall satisfaction with mentorship improved from 57.6% to 73.4% (*p* = .53), satisfaction with emotional support improved from 63.1% to 71.6% (*p* = .21), and satisfaction with career-specific mentorship improved from 48.5% to 59.5% (*p* = .70). Residents reported consistently high satisfaction with peer mentorship (77.8%–100%). The percent of residents reporting they had identified a career mentor increased from 60.0% in 2017 to 88.9% in 2019, which was sustained at 90.0% in 2020.

**Conclusion:**

We report our experience in implementing and adapting a mentorship curriculum for resident physicians in a single training program, including transitioning to a primarily online-based platform at the outset of the SARS-CoV-2 pandemic. Our results showed a trend towards improvement in resident satisfaction with overall and career-specific mentorship, as well as improved emotional support. Future work is needed using more objective outcome markers among a larger and more diverse group of residents.

**KEY MESSAGES**
- Among resident physicians in a single training program, a mix of mentor–mentee dyads, group-based peer mentoring and a structured curriculum has shown promise in improving resident-reported satisfaction with programmatic mentorship
- While we attempted to adapt the mentorship curriculum to an online platform with the development of the SARS-CoV-2 pandemic, reported satisfaction in overall mentorship and emotional support decreased in comparison to the prior year, an important focus for future work.

## Introduction

Mentorship is a vital component of academic medicine [1–4]. Benefits of a meaningful mentor–mentee relationship include increased trainee productivity [5] and confidence [6], reduced trainee burnout [7], higher career satisfaction [8] and retention in a chosen career [9]. Mentorship has also been shown to impact career choice [10,11]. Yet, challenges persist in developing successful mentorship programs, including lack of clear guidelines, limited academic support and reliance on informal training of mentors [12]. Congruent with those findings, reports have concerningly documented low rates of mentorship within residency training programs and among junior faculty [1,2], with more recent surveys finding only a modest increase in the prevalence of formalized mentorship programs among faculty [13,14].

The recent social distancing necessitated by the SARS-CoV-2 pandemic has exacerbated challenges in identifying and fostering mentor–mentee relationships. Further, what constitutes effective mentorship remains the subject of ongoing debate, with traditional reciprocal dyads between a less-experienced trainee and senior faculty member [2,3] potentially limiting the diversity of perspectives and increasing time demands on mentors. Recent work has emphasized fostering mentoring networks [15,16], the importance of peer-based mentorship [17–19] and the importance of understanding well-characterized mentee [20–22] and mentor [23–28] behaviours to endorse and model or avoid.

Mentorship program development should ideally be multifaceted to address all of those components. Such efforts have been reported successfully for junior faculty [29]. In this commentary, we discuss our experience with the development and iterative adaptation of a mentorship curriculum for resident physicians. Using an observational approach to generate hypotheses for future programmatic iterations, we report promising trends in resident satisfaction after implementation of that curriculum.

## Curriculum structure and method of implementation

The combined Internal Medicine-Paediatrics Residency Program at Brigham and Women’s Hospital and Boston Children’s Hospital is four years in length with an average of four residents at each post-graduate level. As a part of the program, all trainees meet monthly for half-day educational sessions focussed on professional development, community-building, programmatic quality improvement and board review. Annually, current residents provide feedback through an anonymous survey on residency leadership, programmatic support, the training experience, scholarly work including quality improvement and career goals. Beginning in 2012–2013, the survey included questions assessing satisfaction with overall program mentorship, career mentoring, and emotional support on a 5-point Likert scale (Supplementary A). Questions about peer mentorship and identification of traditional career mentors were added later (2015–2016 and 2017–2018, respectively). Anonymized annual survey results from 2012 to 2015 revealed resident dissatisfaction with residents’ ability to identify mentors, optimize mentor–mentee relationships and broaden their professional networks in addition to endorsing a lack of social connection within the residency program [30].

In response to those survey results, the initial iteration of the mentorship curriculum implemented in 2016 utilized a peer-based mentorship model for ease of implementation within a small residency program and prior work demonstrating notable benefits among junior faculty [31]. While multiple modalities of peer mentorship exist [32], we began with a “buddy” model, pairing senior residents with intern and junior residents based on professional interests. Time was provided during regular academic half-days throughout the year and the annual retreat for structured discussions, which focussed on the exchange of practical advice and the provision of emotional support. However, given constraints on resident attendance during the academic half-days (e.g. residents who were on vacation, global health rotations, or overnight blocks), the buddy model left some residents unpaired during structured time if their partners were not present. Furthermore, it was generally noted that while there were certain advantages to pairing senior residents with junior residents, residents who were in the class directly above could often provide more specific feedback on upcoming rotations, expectations for new clinical roles and so forth.

In response to positive feedback on the curriculum and the aforementioned limitations, the buddy model was replaced with the “family” model in 2017, where one resident from each class was placed in one of four mentorship families, with an attempt made to group residents with similar professional interests wherever possible. Time dedicated to mentorship families was generally increased and an array of instruments using question prompts were developed and iteratively trialled to support small group discussions (see Supplementary B). Residents were at times broken down into pairs within families (e.g. PGY1/PGY2 + PGY3/PGY4 or PGY1/PGY3 + PGY2 + PGY4), reminiscent of the prior model, with questions that were specific to their stage of training. In general, more practical prompts were used to guide discussion when residents were paired with family members in the class directly above them, while more social-emotional prompts were employed when residents were paired with those two or more years ahead of them. Apart from that structured time, mentorship families were encouraged to meet outside of the hospital for smaller group dinners and other social events.

**Figure 1.**
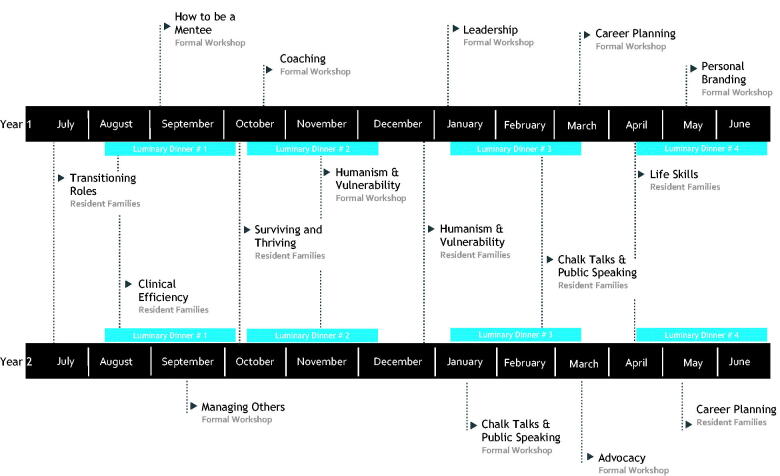
Example timeline of the medicine-paediatrics mentorship curriculum.

The sustained positive reviews of the peer-mentorship model in annual surveys resulted in continued expansion of the program, ultimately providing funding for each resident family to meet outside of residency-scheduled activities. In addition, the scope of the activities included in the mentorship curriculum grew. Under faculty guidance, additional components were added including guest lectures and workshops on mentorship-specific topics. In 2019, the curriculum was formalized (Figure 1) to include 13 core topics based on our review of the literature as well as guidance from senior faculty, which include: being an effective mentee, role transitioning, managing others, clinical efficiency, surviving and thriving, coaching, humanism and vulnerability, leadership, chalk-talks and public speaking, advocacy, life skills, career planning and personal branding. Those topics are covered either in small-group sessions *via* resident families or through in-person/virtual workshops and lectures.

The formalized 2019 curriculum has subsequently grown to include tri-annual 15-minute virtual one-on-one check-ins with program leadership specifically focussed on meeting each resident’s individual mentorship needs. Additionally, we developed a series of evening seminars, or “Luminary Dinners” with accomplished individuals from beyond the residency program to facilitate broader networking and offer exposure to alternate career paths. Finally, we utilize “faculty bridges” or selected faculty members who serve as liaisons with the aim of pairing residents with specific mentors outside the residency program. Parings were based on professional goals and shared interests in order to foster the establishment of the more traditional mentor–mentee dyad. As the typical length of the residency program is four years, we elected to cover the entire curriculum over a two-year period, allowing for one full cycle of repetition in the event of missed sessions.

After the latest iteration of curricular development, we attempted to evaluate the program objectively. We compared resident satisfaction reported on annual surveys during the years before any formal mentorship curriculum was implemented (2012–2015) with resident satisfaction during the years that included the full spectrum of mentorship curricular activities (2016–2021). Satisfaction was defined as responding either satisfied or very satisfied to survey questions (as opposed to neutral, dissatisfied, or very dissatisfied). We used chi-square and fisher’s exact tests to assess statistical association. All analyses were completed in STATA 15.1 (StataCorp, College Station, TX). The Mass General Brigham Institutional Review Board exempted the analysis of de-identified data from institutional review (2021P000979).

## Discussion

**Figure 2.**
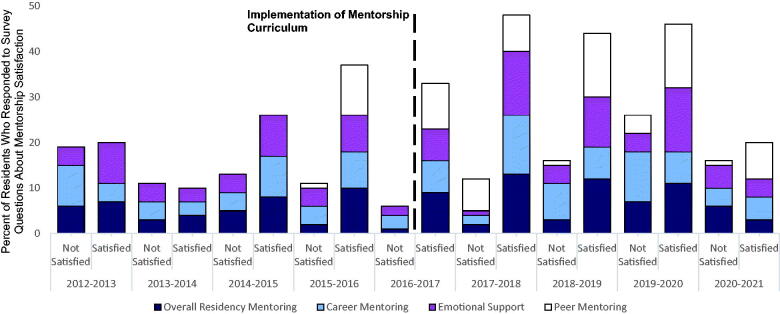
Resident-reported satisfaction with mentorship between 2012 and 2021.

Curriculum implementation was met with anecdotal success, with residents reporting personal improvement in personal satisfaction or mentor identification. On evaluation of 112 responses between 2012 and 2021, with 45 (40.2%) responses from the comparison period 2012–2015, we observed a trend towards improved resident-reported satisfaction with programmatic mentorship experience from 57.6% to 73.4% (*p* = .53). A similar trend was seen for resident-reported satisfaction with emotional support (63.1% to 71.6%, *p* = .21) and satisfaction with career mentorship (48.5% to 59.5%, *p* = .70). The percent of residents reporting they had identified a career mentor increased from 60.0% in 2017 to 88.9% in 2019, which was sustained at 90.0% in 2020. Residents reported consistently high satisfaction following incorporation of a peer mentorship curriculum in 2016 (range: 77.8%–100%). Figure 2 shows resident-satisfaction score by domain for each year of observation.

Much of the prior work done with regard to improving mentorship has focussed on “mentoring the mentors” [33,34] and developing an institutional culture of mentorship [35]. One potential important contribution of our program is the incorporation of didacts dedicated to training residents in optimal mentee behaviours [20–22] to facilitate increased agency within the mentor–mentee relationships by trainees. In addition to focussed didacts among residents, the increase in the percentage of residents reporting identification of a mentor and satisfaction with career mentorship is encouraging. Prior work aiming to improve mentor–mentee pairing has utilized a “speed dating” model in which residents briefly meet with sequential potential mentors in a controlled environment [36]. Our model, which utilizes dedicated “faculty bridges” to connect residents with potential mentors and small group social networking events may offer a more individualized approach to mentor identification. An important next step will be to evaluate the quality and outcomes of the mentorship provided. A recent report showed that among academic faculty who have access to a mentor, there was notable heterogeneity in quality of the mentorship received [37].

The consistently favourable reviews of the peer-based mentorship component of the curriculum are congruent with current literature emphasising the importance of peer-based mentorship across a variety of settings [17–19]. Specific to residents, we found that peer mentor dyads may be a less successful approach compared to peer groups (or a resident “family” model), in large part due to the limitations in availability imposed by residency schedules. Such an approach is an important alternative to dyads of peer-based mentorship, which have notable limitations including challenges in availability and the potential for competition between residents if resources or awards are a constraint [38–40].

The challenges of the SARS-CoV-2 pandemic made resident in-person-meetings challenging in 2020 and beyond. We utilized the previously dedicated funds to support virtual activities for community-building such as online cooking classes, as well as tasking the heads of each resident family with sustaining within small-group communication consistent with some of the aforementioned best-practices for peer-dyad mentorship [38,39]. However, in the year after the SARS-CoV-2 pandemic began (2020–2021), compared to the preceding year (2019–2020), resident satisfaction in overall mentorship decreased from 61.1% to 33.3% (*p* = .24). Reported satisfaction with emotional support also decreased from 93.3% to 26.7% (*p* = .11), while satisfaction with career mentorship increased from 39.9% to 55.6% (*p* = .45). Thus, it is clear that the sense of community within the residency program suffered throughout the pandemic, and the adaptations though potentially helpful were insufficient. It is difficult to determine with the limited data to what degree increased rates of burnout related to the pandemic [41,42] contributed to those changes, but further steps to foster small-group communities despite social distancing could have been helpful in mitigating the effect of the pandemic on the larger community. Such steps may include virtual game nights, virtual medicine trivia, and virtual resident round tables to discuss the state of the program and ongoing curriculum development. That approach borrows from the success of online mentoring (or e-mentoring), which has used similar techniques in other arenas [43–45].

One component that was likely integral to the success of our mentorship program was the development of the curriculum within existing systems (such as the protected time through our academic half-day sessions and annual retreat), which supported ongoing informal peer relationships. Furthermore, by incorporating diverse faculty as “faculty bridges” and as Luminary Dinners invited guests, the curriculum directly addressed two resident-identified needs: help in identifying mentors and access to a broader network. Promisingly, all components of this curriculum are easily implemented in other settings.

It is important to note that the findings of this study are observational in nature and from a small representative sample where the proportion of residents that responded to annual survey questions varied (41.2–100%), which limits our power to identify statistically significant changes. An additional limitation of this study is the lack of an objective outcome measure or measure of mentorship success in the personal and professional domains. An important subsequent step for programmatic evaluation and a consideration for other programs planning to implement similar curricula are to evaluate more objective outcome measures such as resident publications, fellowship match results, subsequent career satisfaction and academic promotion advancement, and levels of burnout. Further, focus groups with residents who have participated in the curriculum will be important for iterative development of individual components of the curriculum. Finally, what constitutes effective mentorship is likely to be highly individualized, and an understanding of the socio-demographic backgrounds of residents participating in such a curriculum may provide insight into how residents’ backgrounds affect their engagement and reception of the curriculum; we were not able to collect socio-demographic data on respondents given that the annual resident surveys are anonymous. We do hope, however, that sharing our preliminary results and lessons learned from our experience in implementing this curriculum will inform future efforts to promote mentorship in academic medicine.

## Conclusions

We report our experience in implementing and adapting a mentorship curriculum for resident physicians in a single training program *via* a mix of mentor–mentee dyads, group-based peer mentoring, and a structured mentorship curriculum. Our experience suggests a trend towards benefit in resident-reported satisfaction with various components of mentorship; however, the social isolation consequent to the SARS-CoV-2 pandemic has presented new challenges. Further, our findings are observational, and further work is necessary to fully assess the impact of such a curriculum using more precise outcome markers, ideally among a larger and more diverse resident group.

## Supplementary Material

Supplemental Material
