# Supplementary material for: Implementation and first experiences with a multimodal mentorship curriculum for medicine-paediatrics residents
Source: Ann Med. 2022 May 11;54(1):1313–9. doi: 10.1080/07853890.2022.2070661 (PMC9103397; doi:10.1080/07853890.2022.2070661)
Supplement: Supplemental Material [file IANN_A_2070661_SM9931.zip › Supplemental files/AllanBlitz_SupplementA_020522.docx]

**Supplement A: Annual Survey of Resident Satisfaction with Mentorship**

| **Question 1: How Satisfied are you with the following:** | Overall Residency Mentoring | Small-group Peer Mentoring Initiative | Career Mentoring | Emotional Support |
| --- | --- | --- | --- | --- |
| Very Dissatisfied |  |  |  |  |
| Dissatisfied |  |  |  |  |
| Neither Satisfied nor Dissatisfied |  |  |  |  |
| Satisfied |  |  |  |  |
| Very Satisfied |  |  |  |  |
|  |  |  |  |  |
|  |  |  |  |  |
| **Question 2: Do you have one or more career mentors?** |  |  |  |  |
| Yes |  |  |  |  |
| No |  |  |  |  |
