# Supplementary material for: Implementation and first experiences with a multimodal mentorship curriculum for medicine-paediatrics residents
Source: Ann Med. 2022 May 11;54(1):1313–9. doi: 10.1080/07853890.2022.2070661 (PMC9103397; doi:10.1080/07853890.2022.2070661)
Supplement: Supplemental Material [file IANN_A_2070661_SM9931.zip › Supplemental files/AllanBlitz_SupplementB_020522.pdf]

DIVING DEEPER

Rules:

- 10 minutes per round, with 2.5 minutes per person each round to respond. Rotate the order in which each person responds with every round.
- Choose one question from the row for the round. Each question can only be answered once. Cross it out when it is chosen (everyone should have at least some choice in what they are answering, since there are only 4 per group).
- No interrupting while someone is speaking, even to ask clarifying questions or respond empathically.

Round 1	Share an obstacle that you have encountered as an adult, how it affected you at the time, and how you coped with it or overcame it.	Share a time when you felt disrespected by someone you were working with. How did you react? How would you react today?	Share the story of someone in your life who's had the greatest impact on your decision to become a doctor – how did they shape you?	Share a story about someone who believed in you and who mentored you in some capacity – how did their support affect you?	What kind of a kid were you growing up? How did your childhood shape you as an adult? How have you changed (or not changed)?
Round 2	Talk about your high school experience. Did you enjoy high school or hate it? Who did you hang out with? What activities did you do?	How did you choose where to go for college? What did you focus on during your time as an undergrad? How did you spend the summers?	Share a story about someone you became close to who was especially different from you or challenged your preconceived notions.	What period of your life do you feel you grew the most in as an individual? What prompted that growth? What were the lessons learned?	What is your favorite book? When/why did you first read it? Why were you so drawn to it? How did it change the way you think about the world?
Round 3	Talk about a time when you doubted your decision to go into medicine. What brought that doubt on? How did you overcome it?	What do you value most in your relationships with others? Has that changed over time? How do you nourish your friendships?	What have been the major forces that have shaped your moral compass and sense of justice? What are your guiding moral principles?	Talk about a time when you let someone down or didn't do the right thing. How would you approach the situation differently today?	How do you think about your identity? What are the affiliations that mean the most to you? How has your sense of identity evolved?
